# Advanced statistical models to handle response styles and uncertainty when modelling emotional intelligence of elite swimmers

**DOI:** 10.1038/s41598-026-46938-4

**Published:** 2026-05-22

**Authors:** Moritz Berger, Rosa Fabbricatore, Maria Iannario, Gunther Schauberger

**Affiliations:** 1 Core Facility Biostatistics, Medical Faculty Mannheim, Central Institute of Mental Health, Heidelberg University, Mannheim, Germany; 2 Department of Economics and Statistics, University of Naples Federico II, Naples, Italy; 3 Department of Political Science, University of Naples Federico II, Naples, Italy; 4 TUM School of Medicine and Health, Chair of Epidemiology, Technical University of Munich, Munich, Germany

**Keywords:** Human behaviour, Statistics

## Abstract

Emotional intelligence is a key factor for success in sporting competitions, arousing great interest in the psychological assessment of athletes. When the evaluation of psychological behaviour relies on Likert-type psychometric scales, individuals could tend to respond to items regardless of their content or by selecting the extremes or the middle part of the response scale, compromising the measurement process. In this vein, the present paper aims to address measurement issues regarding uncertainty and response style during the assessment of emotional intelligence of elite swimmers by exploiting latent trait models. Results provide evidence in favour of models accounting for specific response behaviour compared to simple item response theory models.

## Introduction

Data concerning athletes’ performance and behaviour are essential requirements for competitive sports, as they support tailored actions to optimise training, improve competition strategies, and safeguard athletes’ health^1–3^. Recently, increasing attention has been devoted to understanding the psychological functioning of athletes and how personality traits influence their performance^4–6^.

Among these traits, *emotional intelligence* (EI)^7–9^ has emerged as a central construct, referring to the ability to perceive, regulate, and effectively manage emotions in competition, thereby supporting optimal performance^10^. Athletes with high EI are generally better equipped to cope with stress, manage conflicts, and inspire others. One of the most influential conceptualisations of EI, proposed by Petrides and Furnham^11^, identifies four core dimensions: *Well-Being*, *Self-Control*, *Emotionality*, and *Sociability*. In athletic contexts, each dimension plays a distinct role depending on the nature of the sport. A recent study^12^, for example, highlights the predominant role of the Self-Control dimension in competitive environments. As detailed in the motivating example, Self-Control—which encompasses emotion regulation, stress management, and low impulsiveness—is particularly crucial for athletes competing in individual sports without direct opponents, such as swimming, where performance relies heavily on regulating one’s internal emotional states^13^. Given its relevance and its centrality in our empirical analysis on elite swimmers, the Self-Control dimension is therefore the focal point of the present study, which aims to illustrate the application of response-style modelling in psychological trait assessment.

From a modelling perspective, EI can be conceptualised as a latent personal attribute measured indirectly through multi-item psychometric scales. Latent variable models thus provide an appropriate framework for capturing such constructs. When responses are categorical, especially on Likert-type scales, Item Response Theory (IRT) models constitute the main methodological reference^14^. In general, IRT posits that the probability of selecting a given response category depends on: (i) the respondent’s position on an unobserved latent trait, and (ii) item-specific parameters describing, for example, category thresholds. This probabilistic structure enables measurement on a common latent scale and offers major advantages over classical test theory, including parameter invariance and explicit modelling of item functioning.

Among IRT models for polytomous items^15^, the Partial Credit Model (PCM)^16^ is adopted here. The PCM, belonging to the Rasch family, is specifically designed for ordinal items with more than two categories. It provides a coherent probabilistic framework for sequential integer scoring while retaining the Rasch property that the total raw score is a sufficient statistic for the latent trait. Its use is often preferred over the non-conditional Graded Response Model (GRM)^17^ in applications involving unidimensional constructs. Compared to the GRM, the PCM also enjoys favourable mathematical properties, as it does not require imposing specific constraints on the predictor.

It is also well established that item responses may be affected by factors unrelated to the latent trait, particularly when dealing with sensitive constructs. Prior studies^18^ document the presence of response styles, such as a tendency to prefer extreme or middle categories independently of item content. In the present application, such tendencies are likely to manifest in the use of extreme categories when reporting levels of agreement or disagreement. Other research^19^ focuses on response behaviour driven by varying degrees of uncertainty in selecting a preferred category. Ignoring such heterogeneity may lead to biased parameter estimates, motivating the development of latent trait models that extend the basic PCM to incorporate both uncertainty and response styles.

In this study, we analyse extensions of the PCM that account for athletes’ uncertainty and response styles when responding to Likert-type items measuring Self-Control. In particular, we consider the *PCM with Response Style* (PCMRS) and the *Uncertainty PCM* (UPCM). Furthermore, both the underlying trait and the response-style (or uncertainty) components are linked to explanatory variables such as age, sex, and the Big Five personality traits.

### Problem statement

**Fig. 1:**
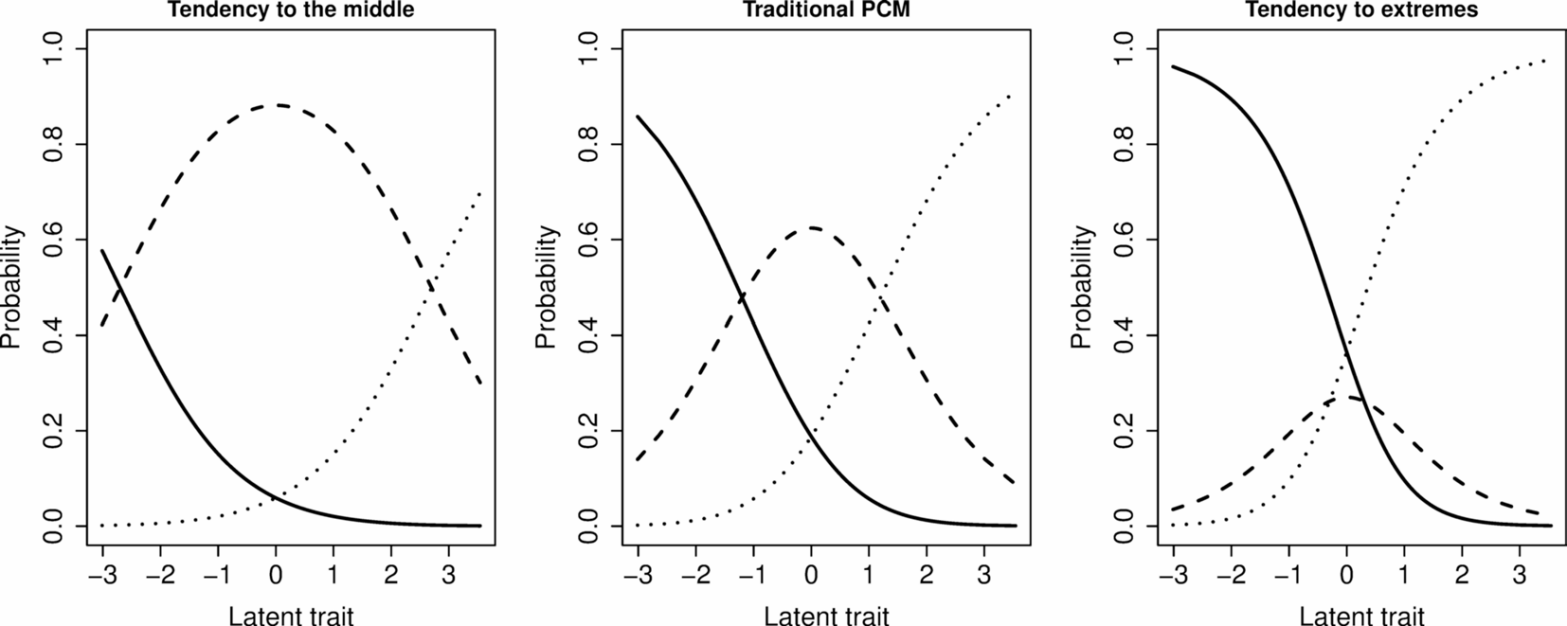
Effect of response styles on category response curves (solid lines: category 1, dashed lines: category 2, dotted lines: category 3). Left: distortion due to tendency to middle categories, Middle: expected category curves under a traditional Partial Credit Model, Right: distortion due to tendency to extreme categories.

In the case study considered here, the distribution of responses across the Likert-type categories shows a clear tendency toward both extreme and middle options. Such patterns indicate the presence of systematic response styles, which cannot be adequately captured by the traditional PCM (see Fig. 1 for an example with three categories). Because the PCM assumes that category transitions reflect only differences in the latent trait, ignoring response styles leads to biased or inefficient parameter estimates. This issue is well documented in the literature: when response tendencies such as extreme responding or midpoint preference are not modelled, the accuracy and interpretability of item and person parameters can deteriorate substantially^18^. For this reason, alternative model specifications that explicitly accommodate response-style behaviour are required.

As mentioned, the term response style refers to an individual’s systematic tendency to select certain categories—such as extreme or middle options—regardless of the substantive content of the item. A related but conceptually distinct phenomenon is uncertainty. Individuals who are confident in their opinion tend to select a specific category with high probability, whereas respondents who lack a definite opinion show high uncertainty and exhibit no systematic preference for any type of response category. Although such behaviour may appear stylistic, it is not a response style in the traditional sense, since it does not reflect a stable preference for particular categories but rather the absence of a clear stance. To avoid confusion with the conventional meaning of response style, we treat uncertainty as a separate [construct see Ref.^20–22^ for comprehensive discussions of uncertainty in rating studies and ordinal data]. Its explicit parametrisation enables the identification of subgroups that differ both in their level of uncertainty and in their underlying latent trait.

The modelling approaches incorporating uncertainty and response styles are illustrated using data from a survey conducted within a joint project between the University of Naples Federico II and the Italian Swimming Federation (Campania Regional Committee).

The survey conducted from 11/11/2019 to 15/12/2019 gathers information about psycho-social aspects influencing elite swimmers’ performance. The authorisation operations were carried out by the Italian Swimming Federation (FIN), which asked in the athletes’ registration form for authorisation to process the data collected. For participants who were minors, consent was provided through the procedures adopted by the sports federation and the affiliated clubs. Respondents were informed that the collected data could be used for research purposes within the scope of the survey. No ethics committee was contacted for the collection of the information that is part of the data collection activities of the athletes carried out regularly by FIN. In accordance with the EU General Data Protection Regulation (Regulation (EU) 2016/679, GDPR), the Italian Data Protection Code as amended by Legislative Decree No. 101/2018, and the “Ethical Rules for Statistical or Scientific Research” issued by the Italian Data Protection Authority (art. 2-quater and art. 106), the use of fully anonymized data for non-clinical research purposes does not require prior approval from an ethics committee. Participation in the study was voluntary and in full compliance with the applicable privacy and data protection regulations. Participants were informed that they could request about any aspect of the study by contacting the research team via the email addresses provided in the study information sheet. In addition, participants were free to discontinue completion of the questionnaire at any time without any consequences. See Iannario et al.^23^ for further information on the study objectives. Therefore, using the data at hand (which was not explicitly collected for the analysis presented here), we set out to answer the following questions:

1. What is the effect of response style and uncertainty on the accuracy of responses measuring Self-Control?
2. What can be inferred about EI profiles by analysing the influence of covariates (i.e., age, sex, and Big Five personality traits) in the context of professional swimming?

### Contribution

By answering the two research questions, we may contribute to the field (1) implementing updated versions of the PCM, namely two different extensions of the basic model that make use of response style and uncertainty in the process of selection of categories by respondents who are often faced with complex questions that affect their psychology; (2) providing indications concerning the relationship between elite swimmers responses, accuracy and characteristics of respondents themselves.

The paper is structured as follows: in Sect. “Motivating example: analysing the psychological profile of elite swimmers”, we provide the background from which this work stems describing the survey data and variable selection. Section “Methods” describes the models used in the analysis, starting from the basic PCM. Section “ Elite swimmers’ response behavior during EI assessment” continues comparing the results of the fitted models and discusses the main results, providing the ground for the conclusion in Sect. “Conclusion”.

## Motivating example: analysing the psychological profile of elite swimmers

It is well established in the literature that personality traits affect athletes’ performance and success in competitive sports^24^. This represents the core interest of a branch of psychology named sports psychology, which aims to explain the performance and well-being of athletes in terms of their psychological traits and mental skills^25^. In this context, researchers typically refer to the well-known theoretical framework of the five-factor model^26^, including the Big Five factors of *Emotional stability*, *Extraversion*, *Openness*, *Agreeableness*, and *Conscientiousness*. Some relevant findings in this area emphasised a positive effect of Extraversion, Conscientiousness, Emotional stability, and Openness on performance^27,28^, whereas a negative association emerged for the Agreeableness trait^28^. However, it is worth noting that several differences emerged according to the characteristics of the discipline (e.g., high-risk, individual, or team sports), which should be considered to obtain more valuable insights. In this regard, an inclusive meta-analysis on the relationship between the personality traits of elite sports athletes and performance can be found in Yang et al.^29^.

Recently, in addition to the Big Five factors, great interest has been paid to EI as another personal trait^30,31^, due to the prevalence of pressure situations in sports. EI refers to the individual responses to intrapersonal or interpersonal emotional information and encompasses the identification, expression, understanding, regulation, and use of emotions^30,32^. The complex nature of EI requires to differentiate among their multiple facets, leading researchers to generate many theoretical conceptualisations embracing multiple dimensions of EI. Based on a content analysis of the salient models proposed in the literature, Petrides and Furnham^11^ identified 15 common facets of trait EI organised in four factors: *Well-Being* (self-esteem, trait happiness, and trait optimism), *Self-Control* (emotion regulation, stress management, and low impulsiveness), *Emotionality* (emotion perception, trait empathy, emotion expression, and relationships), and *Sociability* (assertiveness, emotion management, and social awareness).

EI represents a key factor for success in sporting competitions for different reasons^33^. Firstly, it enables athletes to properly perceive and manage their emotions during competitions, such as fear and anxiety, thereby optimising their performance^13,34^. Additionally, it empowers athletes to recognise, regulate, and use the emotions of both opponents and teammates^13^. A high level of EI is also associated with greater personal satisfaction in performance and improved achievement of season objectives^35^. Moreover, it influences neurophysiological stress responses, including variations in cortisol secretion^35^. Indeed, research has shown that trait EI shapes how individuals interpret and anticipate stressful events: those with higher EI are more likely to perceive stressors as challenges rather than threats, displaying reduced cortisol release in anticipation of a novel and potentially stressful situation^36,37^. Lastly, EI facilitates the adoption of psychological strategies that positively impact performance, such as emotional regulation, goal setting, imagery, and self-talk^35,38,39^. As for the Big Five factors, the four dimensions of EI play a different role in determining athletes performance and success according to the characteristics of the sport. Laborde et al.^13^ proposed to differentiate among individual sports without a direct opponent, individual sports with a direct opponent, and team sports. In individual sports without a direct opponent, such as swimming, the ability to perceive and regulate one’s own emotions - particularly anxiety or fear - plays a crucial role in achieving optimal performance. When there is a direct opponent, as in sports like tennis, also the perception and appropriate use of opponent’s emotions become significant for success. Finally, in team sports like soccer, athletes’ ability to perceive, regulate, and use the emotions of their teammates additionally contribute to winning competitions.

Given these premises, the present work focuses on the evaluation of the Self-Control EI facet in a sample of elite swimmers, which is the most relevant EI dimension for individual sports without a direct opponent. Indeed, these athletes are expected to be highly capable of managing pressure and regulating their emotions, which represents the emotional regulation and stress management aspects of the Self-Control EI dimension, because they are almost entirely responsible for a competitive result. Conversely, the ability to perceive and use the emotions of others, namely Sociability and Emotionality EI dimensions, mainly pertain to sports with a direct opponent or team sports.

As typical for psychological constructs, EI is usually assessed through self-report questionnaires. In this paper, the Trait Emotional Intelligence Questionnaire (TEIQue)^9^ was adopted, which has also been validated for sports^35^. However, self-report measures present a non-trivial drawback that consists of the possible presence of inaccurate answers (due to, for example, low attention) and different response behaviours that could affect the assessment quality. In this perspective, this work also addresses measurement issues regarding uncertainty and response style during the assessment of swimmers’ self-control.

In this context, individual characteristics may influence both Self-Control, namely the measured latent trait, and the response behaviour. Notably, age, sex, and the Big Five are considered relevant covariates. Despite the importance of socio-demographic factors, differences in EI by age and sex have not been extensively explored in sports literature^40^. Existing findings suggest a weakly positive correlation between age and EI^41^, attributed to enhanced emotion regulation strategies resulting from accumulated life experiences^42^. Contrasting evidence exists regarding gender differences according to EI dimensions and adopted psychometric scales^35^. Among the studies relying on the TEIQue scale, Mikolajczak et al.^41^ showed a higher global EI score for males, whereas Laborde et al.^35^ reported this impaired results in favour of males only for the Self-Control and Sociability dimensions. Research on the link between trait EI and the Big Five personality traits has generally indicated a positive correlation with EI full-scale and sub-scale scores. Emotional stability and Extraversion have proved to have the strongest association with global EI, while Emotional stability and Conscientiousness primarily influence the Self-Control dimension of EI^43–46^. However, literature examining these relationships in sports remains limited, especially for specific and homogeneous athlete populations such as elite swimmers.

The literature also indicates that age, sex, and the Big Five personality traits are crucial in the assessment of response behaviour. Advancing age is associated with a heightened tendency towards extreme response style^47–49^, possibly attributed to a gradual decline in working memory capacity, rendering respondents more susceptible to response effects^50^. However, findings regarding the gender effect appear inconsistent, with some studies identifying maleness as a predictor of extreme response style while others support an inverse or non-significant effect^47–49,51–54^. Concerning the Big Five, certain scholars advocate for the stability of response behaviour as a trait-like property^49^, shedding light on the relationship between response styles and personality traits. Specifically, there is a broader consensus that extreme response style positively relates to the personality traits of Openness, Conscientiousness, and Extraversion^55–57^. Conversely, results for Emotional stability and Agreeableness have been varied and limited^57^.

## Methods

### Extended partial credit models

Let the categories $\:0,\dots\:,\mathrm{m}$ represent graded agreement/disagreement attitudes with a natural symmetry and $\:\:{Y}_{pi}\:\in\:\:\left\{0,\dots\:,m\right\}\:$denote the response of individual *p* to item *i*. In the partial credit model (PCM) the predictor, when choosing between categories *r* − 1 and *r*, has the form

1 $$\begin{aligned} \log\left(\frac{P(Y_{pi}=r)}{P(Y_{pi}=r-1)}\right)=\eta_{pir} =\theta_{p} - \delta_{ir}\,,\quad r=1,\hdots,m\,, \end{aligned}$$

for $\:p\:=\:1,\dots\:,P$ respondents and $\:i\:=\:1,\dots\:,I$ items. The parameter $\:{\theta}_{p}$ is the person parameter (in our analysis it is referred to the elite swimmer) whereas $\:{\delta}_{ir}\:$ - denoted as the item-step *difficulty* parameter - determines the choice between categories *r* − 1 and *r*. From the predictor of the PCM it follows that the probabilities of adjacent categories are equal if $\:{\theta}_{p}\:=\:{\delta}_{ir}$.

#### Modelling response styles

Response styles are incorporated by modifying the thresholds *δ*_*ir*_ in the PCM. An additional person parameter $\:{\gamma}_{p}$ is inserted into the predictor (1), shifting the thresholds of the response categories of agreement/disagreement in opposite directions.

Let $\:k\:=\:m/2$ denote the middle of the response categories. If the number of response categories is odd (*m* even, as in our analysis) *k* is the neutral middle category between disagreement categories $\:0,\dots\:,k-1$ and agreement categories $\:k\:+\:1,\dots\:,m$.

Then the corresponding partial credit model with response style (PCMRS) proposed by Tutz et al.^18^ has the predictor

2 $$\begin{aligned}\eta_{pir} =\theta_p - \tilde{\delta}_{ir}\,, \quad r=1,\dots,m\,,\end{aligned}$$

with the modified thresholds defined by $\:{\stackrel{\sim}{\delta}}_{ir}\:=\:{\delta}_{ir}\:-\:(k-r+0.5){\gamma}_{p}$. If *m* = 6 (as in our analysis) the categories are divided into {0,1,2} and {4,5,6} and the scaling factors $\:(k-r+0.5)$ result to 2.5, 1.5, *…*,−1.5, − 2.5. This means that the parameter $\:{\gamma}_{p}$ can be seen as a shifting of thresholds, where the scaling factor determines the direction and affects that the differences between the modified thresholds change by a constant. If $\:{\gamma}_{p}>0$, the thresholds $\:{\delta}_{ir}$ of the disagreement categories are shifted to the left and the thresholds of the agreement categories are shifted to the right, indicating a tendency to the middle category. If $\:{\gamma}_{p}<0$, it is the other way round, indicating a tendency to the extreme categories.

As described above, the PCMRS contains not only one but two person parameters, one for the latent trait that is of primary interest and one for the response style. As parts of these person parameters may be explained by person-specific variables, it can be highly interesting and insightful to include person-specific variables into the model. Accordingly, both parameters may include subject-specific characteristics as reported in Schauberger and Tutz^58^. This yields the extended predictor

3 $$\eta_{pir}^* =\theta_{p0} +  {\boldsymbol{ x}^T_{p}} {\boldsymbol{ \beta}}-\tilde{\delta}^*_{ir}\,, \quad r=1,\dots,m\,,$$

where $\:{\stackrel{\sim}{\delta}}_{ir}^{*}\:=\:{\delta}_{ir}\:-\:\left(k-r+0.5\right){\gamma}_{p}^{*}$ and $\:{\gamma}_{p}^{*}\:=\:{\gamma}_{p0}+{\boldsymbol{z}}_{p}^{T}\boldsymbol{\gamma\:}$. Hence, the model contains two sets of subject-specific covariates, where the person parameter for the latent trait is captured by $\:{\boldsymbol{x}}_{p}^{T}\boldsymbol{\beta\:}$ and the response style is captured by $\:{\boldsymbol{z}}_{p}^{T}\boldsymbol{\gamma\:}$. In general, the variables in $\:\boldsymbol{x}$ and $\:\boldsymbol{z}$ can be distinct, overlapping or identical. Given the vast number of parameters to be estimated in the PCMRS with covariates (3) (from now on referred to as PCMRS-X), it is sensible to use a mixed-effects modelling framework and to assume that $\:{\theta}_{p0}$ and $\:{\gamma}_{p0}$ are random effects following a normal distribution, $\:{({\theta}_{p0},{\gamma}_{p0})}^{T}\sim N(0,{\sum}_{RS})$^[61^. The diagonal of the matrix $\:{\sum}_{RS}$ contains the variances of the person parameters and the response style parameters, while the off-diagonal refers to the covariance between person parameters and response styles.

#### Modelling uncertainty

In case of non-contingent response style, which is found if respondents have a tendency to respond to items carelessly, randomly, or non-purposefully, an alternative extended version of the partial credit model accounting for uncertainty is the Uncertainty Partial Credit Model (UPCM). The model explicitly aims at modelling the heterogeneity in the population that may result in strongly misleading parameter estimates in the simple PCM when it is neglected^19^. Here, the common predictor in the PCM (1) is replaced by

4 $$\eta_{pir} =e^{\alpha_p}\,(\theta_{p}- \delta_{ir})\,, \quad r=1,\dots,m\,,$$

which contains the additional subject-specific parameter $\:{\alpha}_{p}$. The latter can be seen as modelling the subject-specific decisiveness or discriminatory power. For large $\:{\alpha}_{p}$, the person has distinct preferences for one of the two categories *r* − 1 and *r*; for small $\:{\alpha}_{p}$ the respondent tends to select one of the possible categories randomly. This is commonly referred to as *uncertainty effect*. More specifically, for ordered thresholds $\:{\delta}_{ir}\le\:{\delta\:}_{i,r+1}$, it follows that: (*i*) if $\:{\alpha}_{p}\:=\:0$, the basic PCM is obtained; (*ii*) for decreasing values of $\:{\alpha}_{p}$, one comes closer to a uniform distribution across categories, whatever the parameter $\:{\theta}_{p}$ is (random responding); (*iii*) for increasing $\:{\alpha}_{p}$, the selection for categories becomes very unambiguous depending on the value of $\:{\theta}_{p}$. A large amount of literature is devoted to the study of this uncertainty effect, starting with the single-item case^22,59^, among others. Furthermore, the UPCM could be generalised with an additional item-specific slope parameter $\:{a}_{i}$ as in the generalised partial credit model introduced by Muraki^60,61^.

As in the PCMRS-X (3) above, subject-specific characteristics may also be included in the UPCM yielding the extended predictor

5 $$\eta^*_{pir} =e^{\alpha^*_p}\,(\theta_{p0} + {\boldsymbol {x}^T_{p}} {\boldsymbol {\beta}} - \delta_{ir})\,, \quad r=1,\dots,m\,,$$

where $\:{\alpha}_{p}^{*}\:=\:{\alpha}_{p0}+{\boldsymbol{z}}_{p}^{T}\boldsymbol{\alpha\:}$. The variables in $\:\boldsymbol{x}$ (determining the location of the latent trait of interest) and $\:\boldsymbol{z}$ (determining uncertainty) can be distinct, overlapping or identical. Again, it is assumed that $\:{\theta}_{p0}$ and $\:{\alpha}_{p0}$, follow a normal distribution, $\:{({\theta}_{p0},{\alpha}_{p0})}^{T}\sim\:N(0,{\sum}_{U})$, within a mixed-effects modelling framework. The diagonals of the matrix $\:{\sum}_{U}$ contain the variances of the location parameters and the uncertainty parameters while the off-diagonal refers to the covariance between location and uncertainty effects. Details on these issues are reported in the appendix of Tutz and Schauberger^19^.

## Estimation of model parameters and inference

Both models, the PCMRS and UPCM, whose predictors are in (3) and (5), can be fitted by Marginal Maximum Likelihood.

(MML) estimation. While $\:{\theta}_{p0}$ and $\:{\gamma}_{p0}$ or $\:{\alpha}_{p0}$ are assumed to be random effects, the item parameters $\:{\delta}_{ir}$ and the regression coefficients $\:\boldsymbol{\beta\:}$ and $\:\boldsymbol{\gamma\:}$ or $\:\boldsymbol{\alpha\:}$ are treated as fixed effects. To effectively use MML estimation, it is beneficial to represent the responses $\:{Y}_{pi}\in\:\left\{0,\:1,\dots\:,m\right\}$ using indicator variables, that is $\:{y}_{pir}\:=\:1$ if $\:{Y}_{pi}\:=\:r$ and $\:{y}_{pir}\:=\:0$ otherwise. These indicator variables allow to represent the responses in the vectorized format.

$$Y_{pi}=r \hspace{0.25cm} \Leftrightarrow \hspace{0.25cm} \boldsymbol{Y\!}_{pi}=(y_{pi0},\hdots,y_{pi,r-1},y_{pir},y_{pi,r+1},\hdots,y_{pim})=(0,\hdots,0,1,0,\hdots,0)\,.$$

The vector $\:{\boldsymbol{Y\!}}_{pi}$ contains a single ‘1’ entry. Using these indicator variables, the marginal likelihood of the PCMRS can be expressed by

6 $$L(\boldsymbol{\beta}, \boldsymbol{\gamma}, \boldsymbol{\Sigma}_{RS};  \boldsymbol {x},  \boldsymbol {z})=\prod_{p=1}^{P}\int\prod_{i=1}^{I}\prod_{r=1}^{m}P(Y_{pi}=r| {\boldsymbol {x}_p},  {\boldsymbol {z}_p})^{y_{pir}}f(\theta_{p0},\gamma_{p0})\,d\theta_{p0}\,d\gamma_{p0}\,,$$

where $\:f({\theta}_{p0},{\gamma}_{p0})$ represents the density of the bivariate normal distribution with mean zero and variance-covariance matrix.

$\:{\sum}_{RS}$. The probabilities $\:P\left({Y}_{pi}\:=\:r|{\boldsymbol{x}}_{p},{\boldsymbol{z}}_{p}\right)\:$are obtained by

$$P(Y_{pi}=r|{\boldsymbol {x}_p}, {\boldsymbol {z}_p})=\frac{\exp\left(\sum_{l=1}^{r} \eta^*_{pil}\right)}{\sum_{s=0}^{m}\exp\left(\sum_{l=1}^{s} \eta^*_{pil}\right)}\,,\quad r=1,\hdots,m\,,$$

with the convention $\:{\sum}_{l=1}^{0}{\eta}_{pil}^{*}\:=\:0$. For the UPCM the marginal likelihood $\:L(\boldsymbol{\beta\:},\boldsymbol{\alpha\:},{\sum}_{\boldsymbol{U}};\boldsymbol{x},\boldsymbol{z})$ is formulated analogously.

For maximisation of the marginal log-likelihood (6) within the mixed-effects modelling framework an appropriate strategy as suggested by Tutz and Schauberger^19^ is to use numerical integration by Gauß-Hermite quadrature that replaces the integral by a finite sum. The latter is then maximised using direct or indirect methods (e.g. the Newton-Raphson algorithm for the first and the EM algorithm for the latter). Alternatively, a Bayesian approach based on Markov Chain Monte Carlo methods or a quasi-likelihood approach may be considered. Details about estimation methods are in Rijmen et al.^62^.

Both for PCMRS and UPCM, implementations for the statistical software R^63^ exist in the add-on packages **PCMRS**^64^ and **UPCM**^65^, respectively. Only **UPCM** allows for an integration of covariates while **PCMRS** is restricted to models without covariates. However, the extension of the PCMRS by including covariates can be seen as a special case of the more general model for response styles in multivariate ordinal response models proposed in Schauberger and Tutz^58^. While this general framework also allows for models using a cumulative link function, here we focus on the special case of the adjacent categories link function yielding PCM-type models. Therefore, in our application, we make use of the corresponding implementation in the add-on package **multordRS**^66^ for fitting the extended models with and without covariates. To allow for comparability of the estimates from **UPCM**, **PCMRS** and **multordRS**, the signs of the parameter estimates from **multordRS** are reversed in our results.

For the selection between models we consider the Bayesian Information Criterion (BIC)^67^ and the Akaike Information Criterion (AIC)^68^. These criteria are defined as:

$$BIC = - 2\log L(\boldsymbol \Theta) + \log(n) par \qquad \mathrm{and} \qquad AIC = - 2\log L(\boldsymbol \Theta) + 2 \, par\,,$$

where $\:L\left(\boldsymbol{\Theta\:}\right)$ is the maximum value of the likelihood of the model of interest, and *par* is the number of estimated parameters in the corresponding model. The smaller the BIC or AIC index, the better the model fit. Therefore, among competing models, the one selected is the model with the smallest BIC or AIC value. Since the BIC has favourable properties for identification of the correct model while the AIC typically exceeds the BIC in terms of prediction, our main analysis will be based on the BIC. Moreover, we will use both BIC and AIC for a robustness check of our results using a resampling approach.

## Elite swimmers’ response behavior during EI assessment

The data analysed in this paper consist of information on 205 elite swimmers enrolled in the Italian Swimming Federation (Campania Regional Committee). Participants were predominantly male (61%) with age ranged between 14 and 32 (mean = 17.8; sd = 4.6). As described above, the athletes completed a questionnaire on EI following Petrides^9^, which is based on 30 items, each measured on a Likert scale (from ‘strongly disagree’ to ‘strongly agree’). The questionnaire measures the four dimensions of Well-being, Sociability, Emotionality, and Self-Control. In what follows, we restrict ourselves to the Self-Control subscale (consisting of six items), which is a very important trait for athletes in individual sports like swimming (see Sect. “Motivating example: analysing the psychological profile of elite swimmers”). The corresponding item description and response distribution are shown in Table 1. It also contains the outfit and infit statistics^69^ of the items obtained from fitting the basic PCM. These statistics indicate some overfit but no underfit of any of the items.

**Table 1:**
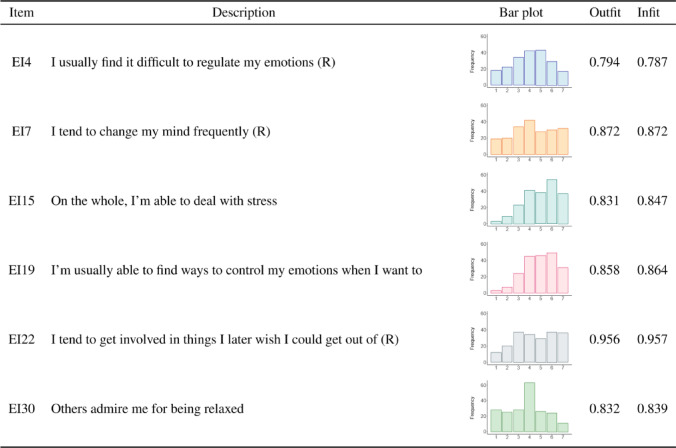
Item description, response distribution, and item fit statistics (Outfit, Infit) based on the PCM for the Self-Control EI sub-scale. (R) indicates reversed items.

**Fig. 2:**
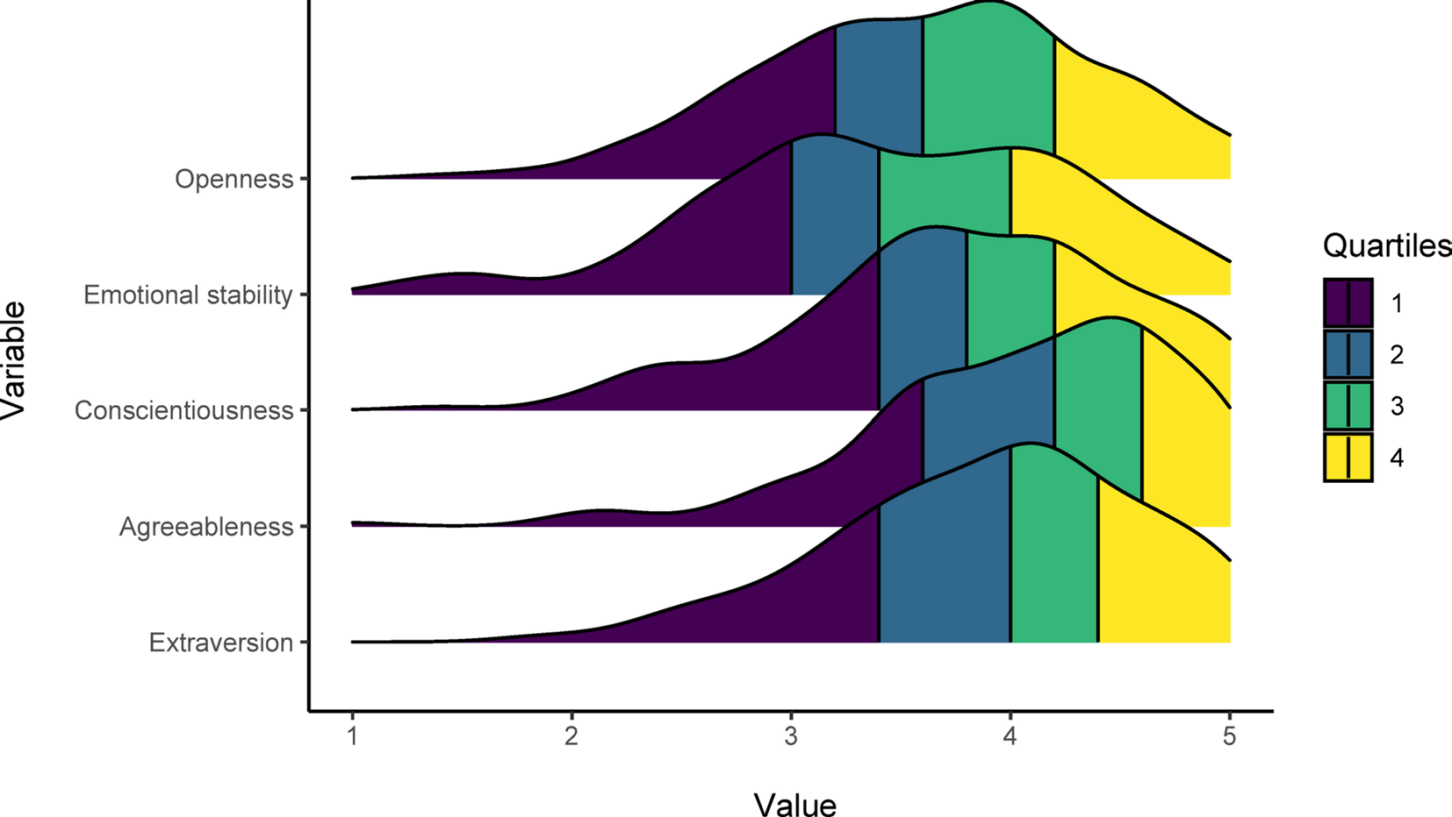
Density ridge plot of the score for the big five personality traits. Colours indicate quartiles.

In addition, the Big Five factors were assessed by using the scale proposed in Barbaranelli et al.^70^ and Caprara and Perugini^71^. Accordingly, swimmers were asked to indicate how each of the 25 adjectives in the list is appropriate for describing themselves on a 5-point scale (not at all, slightly, moderately, quite a lot, at all). The score of each sub-scale (Big Five factors) is obtained by averaging the score observed on the corresponding items, resulting in a theoretical range from 1 to 5. Figure 2 shows the related density ridge plot.

Before we analyse the data under the consideration of person-specific covariates, we start off by analysing the data with a basic PCM (1) and its extensions UPCM (4) and PCMRS (2), which adapt for different types of response styles or uncertainty among the participants.

The variance of the random effect for the trait parameters in the PCM was estimated to be $\:{\sigma}^{2}\:=\:0.154$. When fitting the PCMRS and UPCM without covariates, the following covariance matrices resulted:

$$ \hat{\boldsymbol\Sigma}_{RS}= \begin{pmatrix}0.166 & 0.006 \\0.006 & 0.167\end{pmatrix},\qquad \hat{\boldsymbol\Sigma}_{U}=  \begin{pmatrix}0.138 & 0.056 \\0.056 & 1.804\end{pmatrix}. $$

The covariance matrices report the estimates for the variance of the trait and response style (or uncertainty) effects on the main diagonal and their covariances on the off-diagonal. For a comparison of the model fit, we inspect the BIC and the AIC for all three models (compare Table 2). According to Table 2, both models, which reflect either the uncertainty or the response style of the participants, improve the BIC and the AIC compared to the basic PCM. These results highlight the significant influence of response behaviours in swimmers’ answers to EI items and underscore the importance of using IRT models that account for response style and uncertainty when assessing athletes’ psychological traits. Such models allow for a more accurate estimation of the underlying EI construct by disentangling true trait levels from systematic response tendencies. More precise trait estimates enable coaches and sport psychologists to interpret EI scores more effectively, monitor changes over time with greater reliability, and avoid misinterpretations driven by response bias.

**Table 2:** AIC, BIC, and log-likelihood for the basic models without covariates, i.e. PCM, UPCM, and PCMRS.

	PCM	UPCM	PCMRS
AIC	4567	4386	4359
BIC	4686	4516	4488
Log-Likelihood	−2247	−2154	−2140

**Fig. 3:**
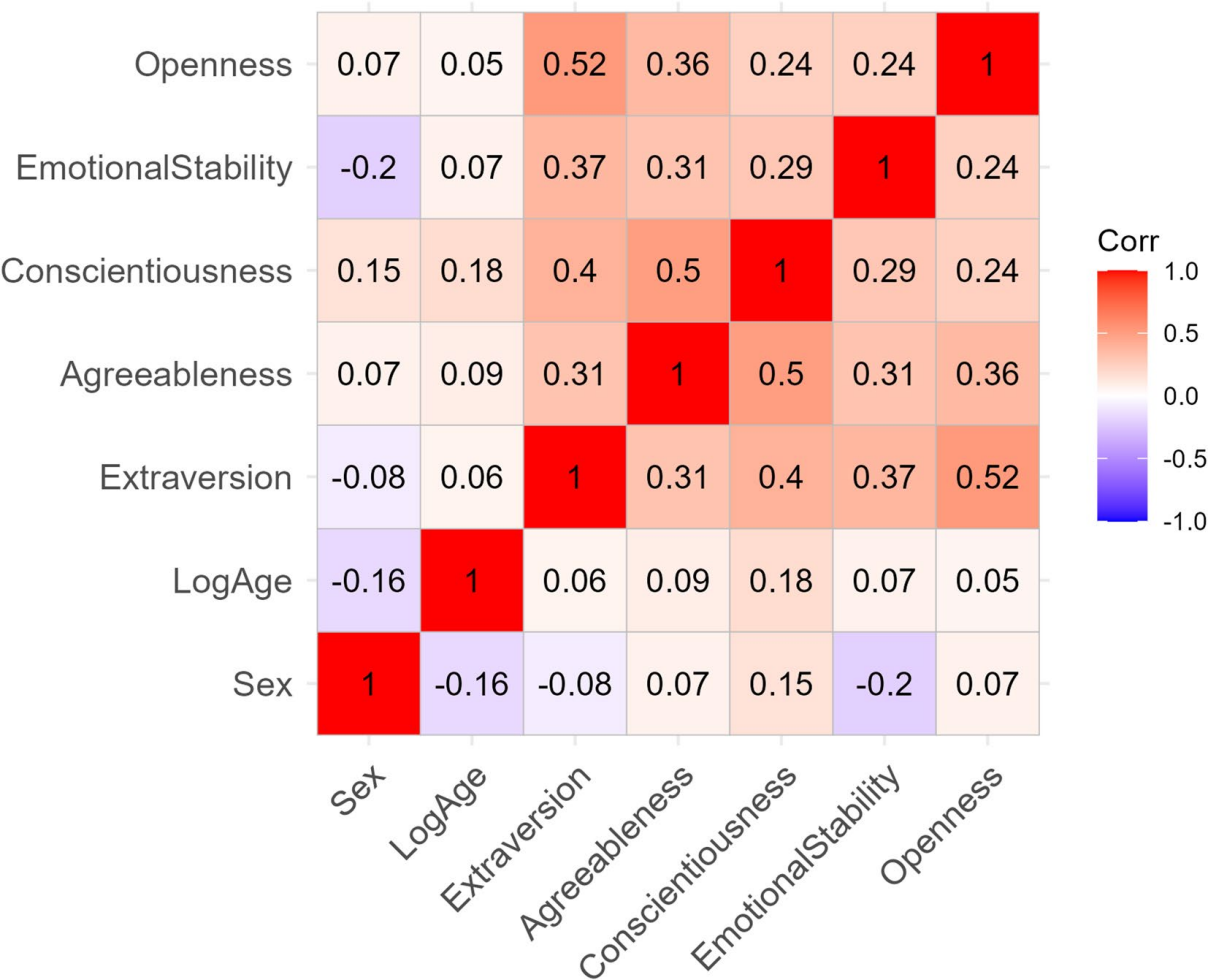
Correlation plot for covariates included in the stepwise selection and the final model.

As PCMRS performs even better than UPCM, we will choose this model as the base model to be extended to PCMRS-X by including covariates. Accounting for individual characteristics within the PCMRS-X helps identify athlete profiles based on both EI levels and response-style tendencies, supporting more accurate assessments and more tailored, athlete-specific psychological interventions. The covariates we integrate into the model are two-fold. First, we use the socio-demographic variables *Age* and *Sex*. Second, we include the Big Five factors (see Fig. 2). The covariates, particularly the Big Five traits, exhibit substantial correlation (see Fig. 3). Particularly high are the correlations between Openness and Extraversion, as well as between Conscientiousness and Agreeableness.

We apply stepwise model selection to the PCMRS-X model using the BIC criterion. The greatest improvement of the model is reached by adding Emotional stability and Conscientiousness to the model. Adding these two variables to the model reduces the BIC to around 4426. All further covariates do not lead to an improvement of the BIC and are, therefore, excluded from our analysis. The corresponding covariance matrix is now estimated to be:

$$ \hat{\boldsymbol\Sigma}_{RS}= \begin{pmatrix}0.104 & 0.021 \\0.021 & 0.130\end{pmatrix}.$$

**Fig. 4:**
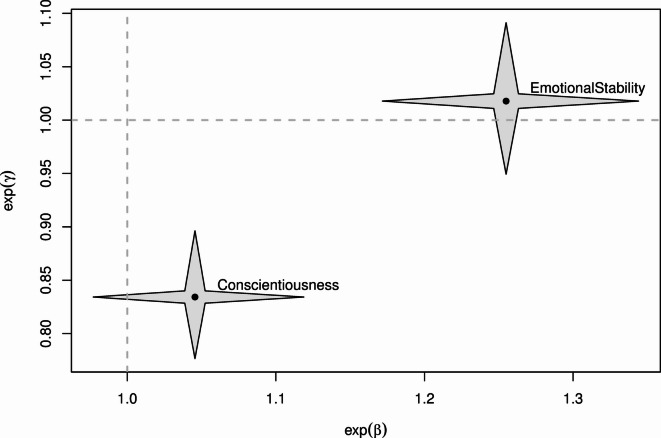
Star plot illustrating parameter estimates and corresponding 95% confidence intervals for covariate effects on location ($\:\boldsymbol{\beta\:}$) and response style ($\:\boldsymbol{\gamma\:}$) in the PCMRS-X model (selected by BIC).

Compared to the previous estimate of the covariance matrix, the variances of both the individual-specific location and the individual-specific response style parameters decreased due to the additional explanatory power introduced by the variables. Figure 4 shows the parameter estimates of the covariate effects used in the final model. The estimates are plotted in two dimensions so that the estimates of the location effect and the response style effect of the same covariate can be shown simultaneously. The stars surrounding the respective points (i.e., point estimates) represent the corresponding pointwise 95% confidence intervals.

As a robustness check of the final PCMRS-X model and the selection process, we applied the model selection process to 100 bootstrap samples of our data. Table 3 contains the selection proportions of all covariates from the 100 bootstrap samples of the original data, separately for stepwise selection with BIC and AIC. The idea of this procedure is similar to stability selection, proposed by Meinshausen and Bühlmann^72^. It can help us to detect whether the selection process is stable and whether BIC and AIC agree in their variable selection preferences. Naturally, the selection proportions according to AIC are higher compared to BIC. Also, the table clearly confirms that Emotional stability and Conscientiousness are the most important covariates. Extraversion and Openness are selected rather frequently by AIC, but rarely by BIC. This might be caused by the rather high correlation between both variables. Overall, the selection by BIC is supported by this sensitivity analysis.

**Table 3:** Selection proportions (in %) of covariates in stepwise model selection for PCMRS-X according to BIC and AIC, based on 100 bootstrap samples of the original data.

	Sex	Age	Extraversion	Agreeableness	Conscientiousness	Emotional stability	Openness
BIC	27	35	19	35	90	100	20
AIC	51	73	77	69	99	100	75

The findings indicate that higher scores on the personality trait Emotional stability are associated with higher scores on the Self-Control subscale. This pattern aligns with prior research^46,73^, which identified Emotional stability as the personality trait most strongly correlated with the Self-Control subscale of EI. Emotional stability includes low anxiety and emotional control, enabling individuals to effectively manage stressful situations and exert self-control^57^. The present study corroborates this association in a sample of elite swimmers. From a practical perspective, this association suggests that athletes with lower scores in Emotional stability may benefit from interventions that incorporate strategies typically associated with this trait, such as mindfulness exercises and stress-management techniques.

Conversely, sex and age did not show a significant effect on Self-Control in the sample under investigation. Regarding sex, the result contrasts with some earlier evidence, such as Laborde et al.^35^, where females showed lower Self-Control scores - an effect the authors attributed to gender-role norms about emotional expression and to more effective stress-coping strategies adopted by men^74^. However, the finding of no sex differences in the Self-Control subscale aligns with more recent evidence from competitive athletes^12^. Regarding age, while some studies support the relative stability of trait EI over time^75^, other evidence suggests that EI can develop with age, change throughout life, and be enhanced through training or targeted interventions^76^. In the present study, the absence of both sex and age effects may be partly explained by the sample’s characteristics. As elite swimmers engaged in highly structured training and preparation programs, these athletes are routinely exposed to similar training demands, emotional regulation practices, and competitive stressors. Such a homogeneous and demanding environment may foster comparable self-regulation capacities across individuals, thereby attenuating potential age- or sex-related differences in Self-Control scores.

In addition, persons with higher scores in Conscientiousness have an increased tendency toward extreme response categories, consistent with previous research^55–57^. The characteristics of conscientious individuals, including intolerance of ambiguity and a sense of certainty, appear to be the primary factors driving this relationship^77^. From an applied perspective, this finding implies that self-control scores observed in athletes high in conscientiousness may not always reflect true underlying trait but, in some cases, a response habit shaped by a need for certainty. Practitioners should therefore consider questionnaire-based assessments with behavioural observations during training or competition, or with coach and informant ratings, to support better-informed psychological interventions.

Finally, sex was not associated with extreme response style, in line with several previous studies^47,51,53,54^. Moreover, the non-significant effect of age on response behaviour may be explained by the relatively young age of our participants (maximum 32 years), which likely mitigated age-related differences in response patterns associated with declines in working memory capacity.

**Fig. 5:**
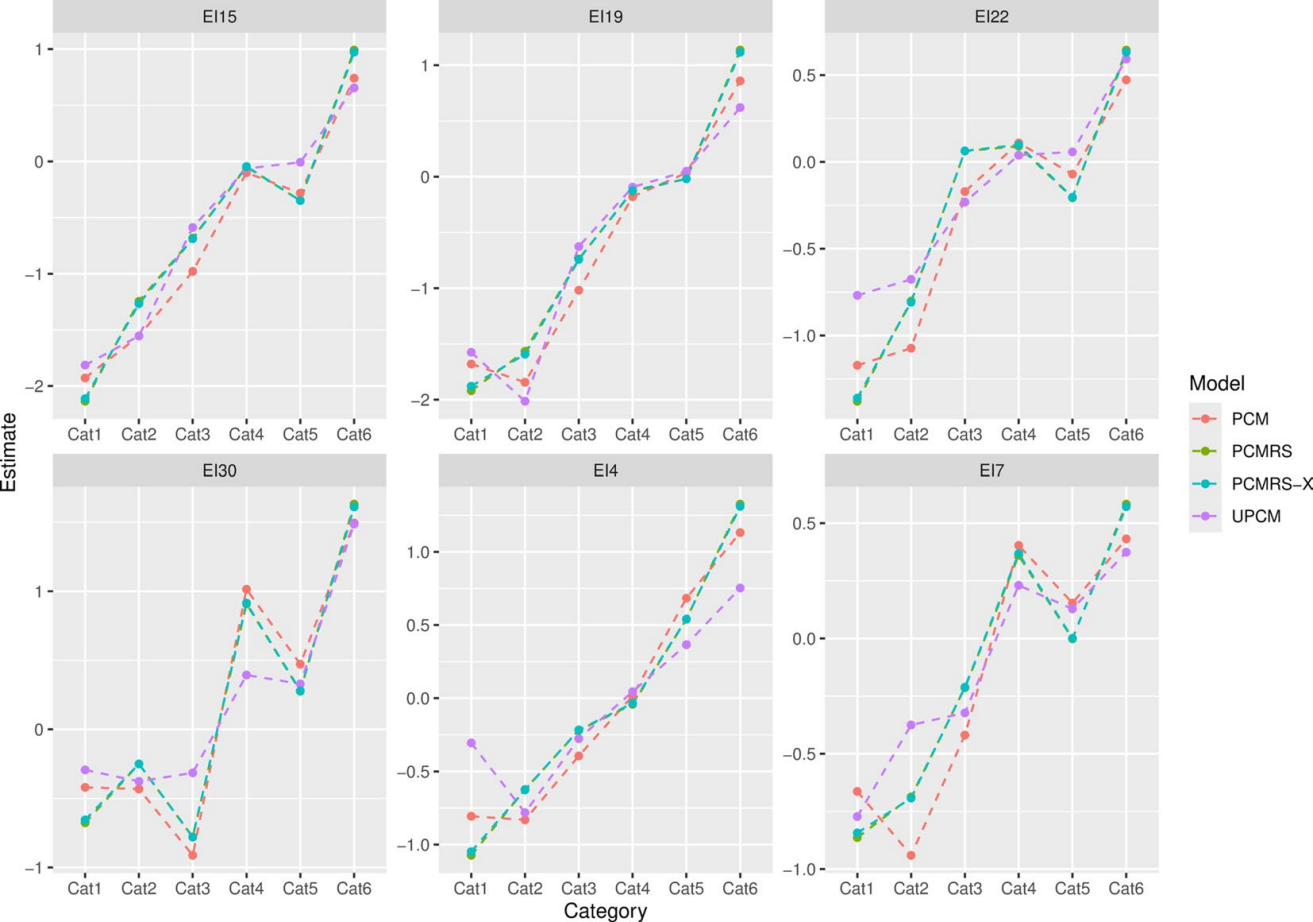
Item parameter estimates for *PCM*,* PCMRS*,* PCMRS-X* and *UPCM*.

Figure 5 displays the estimates of the item parameters for the ordinary PCM, the UPCM, the PCMRS, and the extended PCMRS with covariates (PCMRS-X). For all items, the estimates of the item thresholds differ between the considered models, especially the extreme responses. Between PCMRS and PCMRS-X, the item parameter estimates are nearly identical. However, this is what can be expected because adding covariates to the PCMRS model will mainly affect the estimates for the trait parameters (and the respective covariance matrices) but not the item parameters.

## Conclusion

The study provides some evidence regarding the effect of response style and uncertainty in the assessment of the Self-Control EI subscale of elite swimmers. Improving the accuracy of parameter estimation by exploiting sophisticated statistical models, as those herein employed, allows for disentangling the latent trait component and the response behaviour. These findings hold implications for applied research employing Likert-type measures, such as in personality research, confirming the necessity of accounting for uncertainty and response styles to properly catch the assessed latent construct. Indeed, an accurate measurement of the latent trait undoubtedly represents a valuable step towards the development of effective interventions.

Moreover, accounting for the effect of covariates makes it possible to identify subgroups that differ in response style (or uncertainty) and the underlying trait to better promote successful factors, such as the Big Five, in sports. In this vein, this work provides evidence of the significant effect of Emotional stability on swimmers’ self-control levels. Conversely, the Big Five trait of Conscientiousness is associated with a particular response behaviour characterised by a tendency towards extreme response categories. These findings align with the current literature and offer new insights for sports psychology, specifically regarding EI determinants and the related response behaviours observed during assessment.

Several limitations of the present study should be acknowledged. These refer to possible bias, unmeasured confounding and external validity. First, regarding potential bias, our sample consisted of athletes enrolled by the Italian Swimming Federation (FIN - Campania Regional Committee). Athletes were selected from FIN lists through random selection, and each coach randomly chose one athlete to complete a questionnaire. Although this procedure aimed to minimize selection bias, we cannot completely rule out the possibility of some selection and/or non-response bias affecting our analyses. Second, concerning confounding factors, we modelled uncertainty and response style with respect to socio-demographic variables such as age and sex. However, other unmeasured personal characteristics could still act as confounders, potentially influencing the observed relationships. Finally, with respect to external validity, the study focused specifically on athletes from Italy and the swimming discipline. Therefore, the generalizability of our findings to athletes from other countries or different sports disciplines remains to be established in future research.

Future developments include the possibility of extending the study - again with reference to water sports - but to teams (e.g. water polo team or rowing team) in order to verify possible responses in collective work and training contexts. Further avenues also concern the possibility of working on methodological extensions that take into account more complex criteria for the selection of variables and the possibility of working in the context of a dyad. As already seen in our analysis, the right choice of covariates is essential to get sensible models. For a higher number of covariates, simple methods such as stepwise selection used here may become too time-consuming, so other techniques are in demand. Using penalisation techniques can overcome this issue. For the latter, starting from the paper of Iannario et al.^23^ and Iannario and Karlis^78^ it is possible to approach a study in the subset of athletes whose part of the answers can be compared with that of their coaches analysing the impact of psychological variables such as EI in the confrontation between the parties of the couple.

## Data Availability

The dataset used during the current study originates from regular data collection activities by the Italian Swimming Federation and is available from the corresponding author on reasonable request.
